# Recovery Focused Nutritional Therapy across the Continuum of Care: Learning from COVID-19

**DOI:** 10.3390/nu13093293

**Published:** 2021-09-21

**Authors:** Emanuele Cereda, Pere Clavé, Peter F. Collins, Anne Holdoway, Paul E. Wischmeyer

**Affiliations:** 1 Clinical Nutrition & Dietetics Unit, Fondazione IRCCS Policlinico San Matteo, Viale Golgi 19, 27100 Pavia, Italy; 2 Centro de Investigación Biomédica en Red de Enfermedades, Hepáticas y Digestivas (CIBERehd), Hospital de Mataró, Consorci Sanitari del Maresme, 08304 Mataró, Spain; pere.clave@ciberehd.org; 3 Dietetics & Food Services, Mater Health, Brisbane 4101, Australia; peter.collins@mater.org.au; 4 Mater Research Institute, The University of Queensland, Brisbane 4101, Australia; 5 Circle Bath Hospital, Bath BA2 8SQ, UK; AnneHoldoway@nhs.net; 6 Duke Clinical Research Institute, Durham, NC 27710, USA; Paul.Wischmeyer@Duke.edu

**Keywords:** COVID-19, malnutrition, muscle loss, enteral nutrition, oral nutritional supplement, individualised care

## Abstract

Targeted nutritional therapy should be started early in severe illness and sustained through to recovery if clinical and patient-centred outcomes are to be optimised. The coronavirus disease 2019 (COVID-19) pandemic has shone a light on this need. The literature on nutrition and COVID-19 mainly focuses on the importance of nutrition to preserve life and prevent clinical deterioration during the acute phase of illness. However, there is a lack of information guiding practice across the whole patient journey (e.g., hospital to home) with a focus on targeting recovery (e.g., long COVID). This review paper is of relevance to doctors and other healthcare professionals in acute care and primary care worldwide, since it addresses early, multi-modal individualised nutrition interventions across the continuum of care to improve COVID-19 patient outcomes. It is of relevance to nutrition experts and non-nutrition experts and can be used to promote inter-professional and inter-organisational knowledge transfer on the topic. The primary goal is to prevent complications and support recovery to enable COVID-19 patients to achieve the best possible nutritional, physical, functional and mental health status and to apply the learning to date from the COVID-19 pandemic to other patient groups experiencing acute severe illness.

## 1. Introduction

Patients recovering from COVID-19 disease experience malnutrition [1], low physical function and functional capacity leading to impaired ability to perform activities of daily living [2,3,4]. Long-term consequences of COVID-19 infection include fatigue or muscle weakness even at 6 months after acute COVID-19 infection [4,5]. At follow-up, 60 days after onset of COVID-19 symptoms, over 40% of patients who were hospitalised with COVID-19 report a poorer quality of life [6]. A survey of middle-aged COVID-19 patients who had persistent symptoms, but who were not hospitalised, found that care dependency amongst this group increased [7]. The aetiology of ICU-acquired frailty and weakness is well known, as is the potential for targeted nutrition support to modulate both its development and its treatment [8]. It is likely that patients with severe COVID-19 illness requiring hospitalisation and intensive care will have experienced even more profound physical, functional, psychological and cognitive effects. Nutritional therapy, alongside rehabilitation and physical therapy, has the potential to modify this trajectory. To achieve this, nutritional support must be started early, targeted to individual patient needs and maintained across the continuum of care. We consider how healthcare professionals and policymakers across acute care and primary care can implement assertive, multi-modal, individualised nutritional care that is evidence-based and aims to improve outcomes that matter to patients.

## 2. Focus on the Role of Nutrition in Recovery from the First Day of Hospital Admission 

### 2.1. Multiple Nutritional Challenges Highlight the Need for Early Individualised Nutrition Intervention 

#### 2.1.1. Risk Factors

Following severe COVID-19 disease, malnutrition is common due to the dual contribution of symptoms that reduce nutritional intake and systemic inflammation that drives accelerated muscle loss [1]. A review of three studies that included a total of 589 patients with COVID-19 disease found that, overall, 37% of patients experienced clinically notable weight loss (≥5%) but that 52% experienced weight loss in the study with the highest number of patients who received intensive care, sub-intensive and intermediate care [1]. Anker et al. suggested that, when weight loss reaches clinical significance (≥5%) in combination with impaired functional status and inflammation, COVID-19-related cachexia can be diagnosed [1]. COVID-19 may be a risk for cachexia due to the presence of inter-related features such as weight loss, anorexia, fatigue and raised C-reactive protein levels [9,10]. Screening for the risk of malnutrition using a validated tool should be undertaken, for example, using the ‘Malnutrition Universal Screening Tool’ (‘MUST’) or, for hospitalised patients, the Nutrition Risk Screening 2002 (NRS-2002) criteria [11].

The diagnostic criteria for malnutrition have been recently refined, and the loss of muscle mass is considered an important phenotypic criterion [12]. Malnutrition and muscle loss result from a host of factors, including significant extrapulmonary effects of COVID-19 disease, which make breathing more laboursome, loss of taste and smell reducing the desire to eat in those reliant on oral intake, marked systemic inflammation driving hypermetabolism and muscle catabolism and prolonged periods of bedrest driving disuse atrophy. Up to 40% of patients with COVID-19 experience gastrointestinal symptoms ranging from nausea, vomiting, anorexia and diarrhoea to abdominal distention [13], which can further deter eating and impact on the tolerance to [14], and provision of, nutrition support. 

The nutritional consequences of COVID-19 are not yet fully understood, but the knowledge from severely ill patients with acute respiratory distress syndrome suggests that patients are likely to experience the adverse effects of the loss of muscle mass and skeletal muscle dysfunction for weeks to years. The knowledge from a range of disease states and chronic conditions illustrates that low muscle mass is associated with higher rates of infections, poorer tolerance to chemotherapy, hospitalisation, fractures, reduced quality of life and reduced survival, with implications for patient outcomes and healthcare utilisation [15]. Therefore, the impact of muscle loss during COVID-19 is likely to be profound.

Pre-existing comorbidities such as hypertension, diabetes, cardiovascular disease (CVD) and chronic obstructive pulmonary disease (COPD) are common in patients with COVID-19 and are associated with increased mortality and disease severity [16]. Obesity is also common in COVID-19 patients and has been shown to increase the risk for hospitalisation and poorer outcomes [17]. Obesity may mask malnutrition and muscle loss, but body composition, as assessed by computed tomography, confirms the loss of lean body mass in obese ICU COVID-19 patients, which could lead to the development of sarcopenic obesity [18]. In a recent study of non-critically ill hospitalised COVID-19 patients, obesity (body mass Index (BMI) ≥ 30 kg/m^2^) in combination with two or more comorbidities were associated with increased in-hospital mortality, whereas the presence of obesity with less than two comorbidities appeared to be a protective prognostic factor [19]. 

Oropharyngeal dysphagia (OD) is a further additional risk factor for malnutrition in COVID-19 patients. A recent study with 205 consecutive COVID-19 patients discharged from a general hospital showed that the prevalence of swallowing disorders was 51.7% at admission and still 23.3% at the 6-month follow up, with OD being an independent risk factor for malnutrition [20]. The pathophysiology of OD is multifactorial, arising from neurological factors, respiratory insufficiency, invasive respiratory support, muscular weakness, sarcopenia and cachexia. The loss of taste and smell are common in COVID-19 patients [21], possibly due to the peripheral and central invasive ability of severe acute respiratory syndrome coronavirus 2 (SARS-CoV-2), and may also affect the sensorimotor function of the pharynx and larynx, further compromising the ability to swallow safely [22]. We recommend screening for OD in all COVID-19 hospitalised patients, with the diagnosis of OD made in a timely manner and based on clinical methods such as the Volume-Viscosity Swallow Test (V-VST). Treatment should be focused on compensatory strategies comprising postural management, thickening of fluids and the use of texture modified foods to provide a safe consistency in line with the European [23] and international guidelines [24].

#### 2.1.2. Nutritional Support

The primary focus in the management of COVID-19 patients is to provide respiratory and haemodynamic support. However, there is a need for early, assertive nutrition intervention in critically ill and non-critically ill hospitalised patients to mitigate the symptom, metabolic, nutritional status and nutritional intake factors that contribute to malnutrition, loss of lean body mass and function that, in turn, impair and delay recovery. Indeed, related factors are likely to be present at all stages of the patient journey, as outlined in Table 1. A prospective multi-centre study of non-critically ill hospitalised COVID-19 patients showed that a reduced self-reported food intake before admission to hospital and/or predicted by physicians post-admission was associated with an increased risk of in-hospital mortality or admission to the ICU [19]. In COVID-19 patients in the ICU, achieving a satisfactory caloric intake on day 4 was associated with a lower ICU mortality in a prospective study conducted during the first wave of the pandemic [25].

The role of nutrition—in particular, vitamins and trace elements—in modulating immunity has received much interest during the pandemic. Evaluation of the micronutrient status in a small study of patients hospitalised with COVID-19 disease showed a vitamin D deficiency in 76% of patients and selenium deficiency in 42% but no increase in deficiency in B vitamins, folate or zinc compared to the healthy controls [26]. Cereda et al. 2021 highlighted that, although such observational data supports a link between vitamin D deficiency and COVID-19, it is indirect, and ongoing trials are needed to verify the role of vitamin D in the prevention and/or treatment of COVID-19 [27]. Emerging evidence appears to indicate that high-dose micronutrients (vitamin D in severe COVID-19; single or combined zinc and vitamin C in COVID-19 outpatients) does not confer a benefit on the disease outcome or duration of symptoms [28,29]. The European Society for Clinical Nutrition and Metabolism (ESPEN) recommends that vitamins and trace elements are provided at the recommended daily allowance levels to malnourished patients at risk for or with COVID-19, with the aim of maximising the anti-infection nutritional defence [11]. 

**Table 1:** Symptom, metabolic, nutritional status and nutritional intake factors contributing to malnutrition and impaired/delayed recovery across the continuum of care.

Pre-Acute Illness	Acute Illness	Recovery Phase
Pre-existing noncommunicable disease (NCD): Obesity CVD Diabetes COPD Where chronic inflammation ± reduced cardiometabolic fitness ^1^ contribute to the stress inflammatory response in acute illness Pre-existing loss of body tissue/wasting: Malnutrition Frailty Sarcopenia/Sarcopenic obesity Cachexia (wasting and inflammation)	Effects of acute illness: Stress/inflammatory response Hypermetabolism (increased REE) Increased protein catabolism Bed rest/sedation Oropharyngeal dysphagia GI disturbances disrupting ability to feed Disuse atrophy	Recovery phase complicated by persistent symptoms: Post-intensive care syndrome Functional impairment e.g., fatigue, muscle weakness Oropharyngeal dysphagia Altered appetite and chemosensory dysfunction
**Symptom, Metabolic and Nutritional Status Factors** **↓**		
MALNUTRITION, LOSS OF LEAN BODY MASS AND PHYSICAL FUNCTION CONTRIBUTING TO IMPAIRED/DELAYED RECOVERY		
**↑** **Nutritional Intake Factors**		
Suboptimal dietary quality may already be a concern before onset of acute illness Suboptimal food and nutrient intake linked to NCD [30] Poor diet quality linked to frailty in old age [31] Poor appetite/ability to eat affects physical function [32] Low protein intake linked to reduced strength and physical performance [33]	Nutrient deficits accumulate during hospital stay More than half of patients do not finish their meals in the ward [34] Only 56% of ICU patients meet their requirement for energy ^2^ and 52% for protein [35] Up to 60% of post ICU patients on oral nutrition alone do not meet their energy requirements and up to 70% do not meet their protein targets [36,37,38] Suboptimal use of thickening agents and texture-modified foods for dysphagic patients Patients on texture modified diets have lower energy and protein intake than patients on a normal hospital diet and fail to meet requirements [39]	Ongoing nutritional needs frequently not addressed at discharge Forty-five percent of malnourished patients received inappropriate advice to limit caloric intake [40] Forty-seven percent received general advice that did not address malnutrition [40] Eighty-eight percent received ONS in hospital, but only 6.6% scripted post-discharge [40] Only 11% of HCPs estimated that all patients with COVID-19 were ‘discharged from hospital with a clear nutrition plan’ [41] Suboptimal use of thickening agents and texture-modified foods for dysphagic patients

^1^ The term ‘cardiometabolic fitness’ refers to the presence of insulin resistance, obesity and hypertriglyceridemia rather than physical performance. ^2^ Includes enteral nutrition, parenteral nutrition and propofol. Data presented in the lower part of the table is not specific to COVID-19 patients unless specified. CVD, cardiovascular disease; COPD, chronic obstructive pulmonary disease; REE, resting energy expenditure; ONS, oral nutritional supplements; HCPs, healthcare professionals.

**Figure 1:**
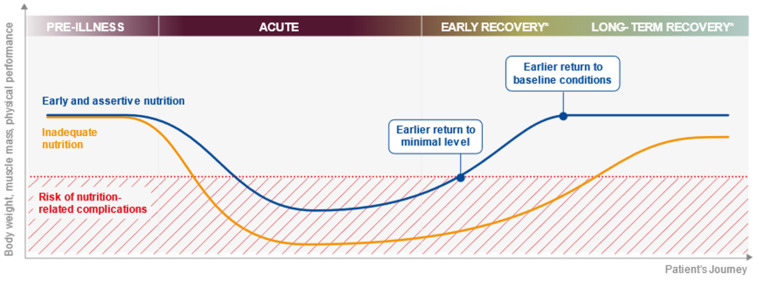
The impact of inadequate nutrition on the body weight, muscle mass and physical performance across the patient journey. * The early recovery period may span step-down care and ward-based care and may last from days to weeks. The long-term recovery phase (post-hospital discharge) may last for weeks or months.

Achieving patients’ nutritional intake targets in hospital (and throughout the patient journey) is challenging during ordinary times (Table 1) but even more so during a pandemic. In hospital, the challenges include a reduced availability of staff or visitors to offer the necessary assistance during mealtimes due to infection control measures; other priorities for healthcare professionals and perceived or real barriers to tube feeding outside the ICU due to oxygen support, e.g., a high-flow nasal cannula, non-invasive ventilation (NIV) such as oxygen masks or the use of continuous positive airway pressure (CPAP). Patients with respiratory failure requiring NIV have been found to often have inadequate nutritional intake [42]. Substantial nutrient intake deficits can accumulate quickly during hospital stay and, without an effective handover, can be overlooked as patients move through different care levels, e.g., ICU, step-down care and ward care. Figure 1 shows the impact of inadequate nutrition on the body weight, muscle mass and physical performance across the patient journey and how early and assertive nutrition has the potential to alter this trajectory.

### 2.2. Enabling Individualised Nutritional Care during Hospitalisation

Practical guidelines for the nutritional management of acutely unwell inpatients with COVID-19 recommend enteral nutrition (EN) in patients unable to meet their nutritional requirements orally with food-based strategies and oral nutritional supplements (ONS) [11,43]. This is particularly important, since it has recently been shown that a reduced food intake in hospitalised non-critically COVID-19 patients is associated with negative clinical outcomes [19]. A pragmatic approach was proposed early in the pandemic, and based on the existing guidance, it provides a protocol for nutritional screening and support in acute care either before ICU admission or during step-down care from ICU stay [44]. If barriers to the use of EN present, the protocol outlines the role of parenteral nutrition. Concerns that the use of EN via nasogastric tubes in patients on CPAP lead to air mask leaks or promote abdominal distention and aspiration due to aerophagia have been expressed; however, practical guidance is available to overcome these issues [45]. All patients who receive NIV should be assessed and managed by healthcare professionals with expertise in nutrition [46]. The aim is to limit the development of malnutrition during hospital stay to enable the optimal recovery after discharge. COVID-19 patients with OD must be prospectively identified, as they may need thickened fluids and texture-modified foods [24].

### 2.3. Enabling Individualised Nutritional Care in ICU

Focused attention on the optimum delivery of nutritional support to minimise nutritional losses, reduce clinical risks and, eventually, facilitate recovery is required on a daily basis across a patient’s healthcare journey from the ICU into step-down care, onto the ward, during rehabilitation programmes and on returning home. This is an essential part of ensuring patients leave critical care as survivors, not victims [47]. Marked and prolonged systemic inflammatory response syndrome (SIRS) is recognised as a hallmark of severe COVID-19 infection [48]. Critical illness polyneuropathy and myopathy are major causes of muscle weakness, which can persist for months or years after discharge and are seen in 56–80% of patients with multiorgan failure with or without SIRS [49].

The guidelines recommend early EN using continuous gastric feeding in patients with COVID-19 disease starting within 24–36 h of ICU admission or within 12 h of intubation and mechanical ventilation [50]. Determining the safe, optimal nutritional needs of ICU patients is key to avoiding the deleterious effects of over- and underfeeding. Predictive equations for estimating the resting energy expenditure (REE) based on the standardised formulae and body weight are imprecise. REE should be measured by indirect calorimetry (IC) [11]. In the context of the pandemic, due to the high number of patients and difficulties with decontamination procedures, it is suggested that IC is performed in all ICU patients after 3 to 4 days [51]. Practical guidance for performing IC during the COVID-19 pandemic is now available [52]. Little is yet known about the effects of COVID-19 disease on REE. 

The first results from a prospective observational cohort study to evaluate the Longitudinal Energy Expenditure and Metabolic Effects in Patients with COVID-19 with respiratory failure admitted to the ICU (LEEP-COVID study) showed progressive hypermetabolism and considerable variations in REE throughout their ICU stay [53]. In the first 3–7 days, the COVID-19 patients were shown to be hypo-to-normo-metabolic (80–100% of predicted, i.e., 17–20 kcal/kg/day). However, after day 7, the COVID-19 patients were hypermetabolic (120–200% of equation-predicted REE even when paralysed (25 ≥ 35 kcal/kg/day)). Therefore, in the first week of ICU stay, it is reasonable to target approximately 20 kcal/kg actual body weight for patients with a BMI of less than 30 kg/m^2^ and adjusted body weight for patients who are obese with a BMI of 30–50 kg/m^2^. To prevent overfeeding, which risks poorer outcomes, one should aim to provide 70% of the estimated or 100% of the measured requirements reached over 4 to 5 days in line with the international expert guidelines and practical guidance for nutrition support in the ICU [8,47]. In combination with other therapies, the careful targeting of nutritional intervention to individual patient REE, as measured by IC, during the acute and post-acute phases of COVID-19 disease has the potential to contribute to the efforts to minimise the effects of hypermetabolism and subsequent development of post-ICU acquired weakness. The recent results from a prospective trial in mechanically ventilated ICU COVID-19 patients suggested that an early caloric deficit (<80% of the estimated requirements on day 4) may independently contribute to reduced survival in the ICU [25].

Patients in the ICU are at a high risk of OD and aspiration pneumonia. Sedative medications, altered states of consciousness and delirium contribute to swallowing impairments [24]. Prolonged orotracheal intubation and tracheostomy are risk factors for dysphagia [24]. Screening for and the assessment of dysphagia are essential in critical care patients [24]. After extubation, the systematic screening and clinical diagnosis of OD should be established, and even instrumental diagnosis, in specific cases [23,24]. Compensatory treatments, including a texture-modified diet, fluid thickening and specific rehabilitation procedures, should be provided, with EN continued until oral intake is sufficient to meet the energy and protein needs [51]. During the acute phase of illness, where catabolic processes are upregulated (e.g., ubiquitin–proteosome pathway [54]) and systemic inflammation is elevated, patients may develop cachexia, and some degree of nutritional losses are inevitable [1]. Although the optimum nutrition strategies for the management of cardiopulmonary cachexia remain unclear, the early initiation of nutrition support that is continued beyond the catabolic phase until the patients are less acutely unwell and more likely to respond to nutritional support will be key. 

## 3. Ensure Continuity of Nutritional Therapy after Discharge

### 3.1. Challenges in Nutritional Care at Discharge

During the current extraordinary challenge posed by the COVID-19 pandemic, it has never been more important to support patients in their recovery. Hospital admission represents an opportunity to identify malnutrition and to implement nutritional therapy. Since hospital stays are increasingly short, the resolution of malnutrition (present before admission or developing during admission) cannot be achieved in the acute care setting. It is therefore paramount that nutritional care is carefully coordinated on hospital discharge. A review of the nutrition support recommendations issued by clinical nutrition professional organisations in response to the pandemic consistently recognised the need for clear pathways from acute care to primary care teams, with accessible and rapid communication links [55]. The lack of appropriate advice and ongoing nutritional care at and after discharge was already a concern [40] and is likely to be exacerbated during the pandemic with the increased numbers of patients and rapid discharge processes.

Three key challenges at this common breakpoint in nutritional therapy exist. Firstly, knowledge of the critical role of nutrition in recovery may be low. Secondly, the timely monitoring of nutritional therapy may not take place. Thirdly, due to pressures on resources, it is common for nutritional care in the community to be delivered by nonexpert practitioners, e.g., general practitioners (GPs) or nurses who follow the standardised protocols designed to provide first-line advice (e.g., fortified food and dietary advice sheets) for a specified period of time—typically 1 month, but in some cases, up to 3 months. The focus is often on selecting a single mode of intervention, e.g., food fortification with a ‘wait-and-see’ approach, with the consequent risk that patients with complex needs, such as patients recovering from COVID-19, may deteriorate whilst waiting for review. Access to and funding for systematic, evidence-based nutritional care, provided by dietitians or nutrition specialists where possible, is needed. A thorough nutritional assessment informs a clear nutritional diagnosis that is communicated and explained to patients and carers. Working collaboratively with patients to understand their perspectives and goals, an individualised evidence-based nutritional care plan is instigated that includes how and when nutritional intervention will be monitored, escalated or de-escalated, as indicated. Clear goals should be established and relevant outcome indicators measured and documented.

Given the high prevalence of obesity and the presence of noncommunicable diseases in those experiencing a severe case of COVID-19, including those hospitalised, it is likely that patients leaving hospital not only require advice on their diet and activity to optimise recovery but also require advice on managing their underlying conditions. Likewise, patients malnourished prior to COVID-19 are at risk of severe COVID-19, as being undernourished compromises immune functions and, therefore, increases susceptibility to infections. The diverse patient group presents challenges in balancing the advice given to both optimise recovery whilst managing underlying conditions such as diabetes. Resources should seek to mitigate the challenges and risk of misinformation by guiding healthcare professionals and patients and carers to the most suitable information based on the stage of recovery, appetite, nutritional status, presence or risk of malnutrition or sarcopenia and presence of comorbidities that are modifiable by diet. Given the complexity, this illustrates the need for dietitians and nutrition experts to be involved in the care pathway.

### 3.2. Enabling Individualised Nutritional Care in Primary Care

Where nutritional therapy is delivered by several healthcare professionals (e.g., GPs, nurses or pharmacists), evidence-based pathways with clear information about goal setting, the selection of appropriate interventions and monitoring is essential to ensure patients receive timely interventions and review to prevent further deterioration in their nutritional status. To support health professionals in primary care in the UK to deliver dietary advice to patients and carers during the COVID-19 pandemic, an existing evidence-based malnutrition pathway was adapted. The COVID-19 Illness Community Support Pathway was developed in conjunction with professional organisations such as the British Dietetic Association (BDA), the Royal College of Nursing (RCN) and the British Association for Parenteral and Enteral Nutrition (BAPEN) and includes patient resources that take account of the patient’s journey [56], for example, recognising that patients who have had an ICU admission are likely to experience ICU-acquired weakness and dysphagia.

### 3.3. The Role of Muscle-Targeted Nutrition in Recovery

COVID-19 survivors are characterised by low physical functioning, reduced functional capacity and an impaired performance of activities of daily life after hospitalisation [3]. Following SARS-CoV infection (during the 2003 global outbreak), impairments persisted for up to 1 to 2 years post-infection in some patients [57]. The factors contributing to muscle loss include immobility, poor nutrition and inflammation, features that are observed in patients with COVID-19 disease. Low muscle strength is suggestive of sarcopenia. The presence of a low muscle quality and quantity confirms the diagnosis, and sarcopenia is deemed to be severe when a low physical performance is detected alongside low muscle strength and low muscle quality and quantity [58]. Sarcopenia has multiple contributing factors, many of which are nutritional, including a low protein intake, inadequate postprandial amino acid availability, decreased muscle response to postprandial anabolic stimuli and vitamin D deficiency. The recommended treatment to target muscle mass and function requires a multi-modal approach with a focus on the optimal protein intake, resistance training and vitamin D supplementation [58,59,60]. The concept of muscle-targeted nutritional supplementation aims to overcome the nutrition-related contributory factors to stimulate the rebuilding of muscle strength and function in patients with sarcopenia.

A recent high-quality trial illustrated what can be achieved when a multi-modal approach was implemented. By combining an individualised physical rehabilitation programme with a muscle-targeted ONS (20-g whey protein, 2.8-g leucine, 800-IU vitamin D and 500-mg calcium) twice daily for 4–8 weeks, compared to a control group who received an iso-caloric protein-free drink, cost-effective improvements were seen for their physical performance, physical function and muscle mass. In those who received the muscle-targeted supplement, a greater proportion were discharged home instead of to a care facility (+24%), had a reduction in intensity of care at discharge (+22%), needed less rehabilitation (−27% in duration) and had a shorter length of stay (−10 days) [61].

## 4. Integrate Individualised, Multi-Modal Nutritional Care across the Patient Journey

**Figure 2:**
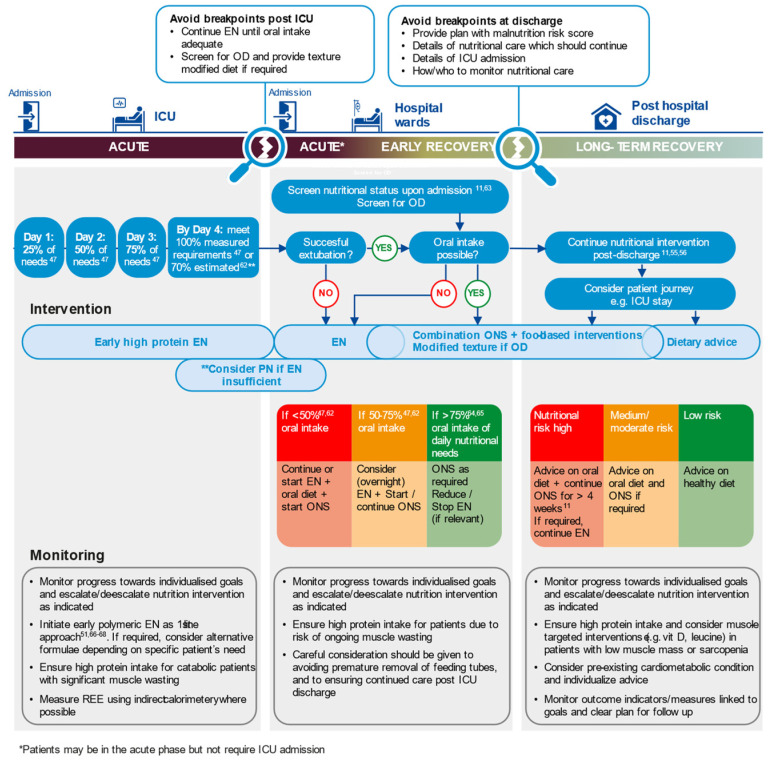
Assertive, multi-modal nutritional care individualised to meet patients’ needs across the continuum of care (see [62,63,64,65,66,67,68]). EN, enteral nutrition; OD, oropharyngeal dysphagia; ONS, oral nutritional supplement; PN, parenteral nutrition; REE, resting energy expenditure.

An approach to nutritional therapy that is applicable across the whole patient journey, from hospital admission, during critical care, into step-down care and onwards, in the community is needed. This should be informed by prospective and universal screening for nutritional risk for all patients on hospital admission and screening for OD in COVID-19 patients. The identification of risk should prompt the provision of basic treatment for all patients at admission to prevent malnutrition. An assessment during hospital admission and at discharge should guide tailored nutritional interventions. Figure 2 shows a visual representation of this approach that can be used to stimulate a knowledge transfer on this topic and is applicable for patient groups beyond COVID-19. The key principles are that nutritional therapy should be:

**Assertive**: Implement early and appropriate nutrition intervention.

**Individualised**: Target nutrition intervention to patients’ individual needs and goals.

**Multi-Modal**: Use a combination of nutrition interventions (see Table 2) to achieve nutritional targets promptly. Avoid single step-up approaches than can lead to delays and further nutritional deterioration.

**Implement across the Continuum of Care**: Coordinate and communicate nutritional care plans across healthcare settings. Monitor progress towards agreed-on goals using appropriate, validated outcome indicators and escalate/de-escalate nutrition therapy as indicated.

Given the current demands on healthcare systems, there is a need to promote self-screening for malnutrition and reduced physical performance to provide easy-to-access, reliable nutrition information focused on recovery for patients and carers and to explain to patients how to monitor their progress not just in terms of weight gain but through simple activity-based functional or performance measures as part of a sarcopenia assessment [58], i.e., return to usual activities, sit to stand test and/or walking test.

## 5. Learning from COVID-19

Stark evidence exists that demonstrates the negative impact of COVID-19 on nutritional status, functional capacity and physical performance. Early, assertive and individualised nutritional care offers the capacity to alter the trajectory to recovery. The prevalence and burden of OD and malnutrition in patients hospitalised in COVID-19 wards is very high [20]. Optimising the management of malnutrition might shorten the hospitalisation period, but optimising the management of OD will likely impact the nutritional status of COVID-19 patients and improve their clinical outcomes and survival after hospital discharge. The COVID-19 pandemic has brought existing key challenges in nutritional care under the spotlight but offers a stimulus to embed new practices, developed in quick response to the pandemic, into routine practice to ensure that patients receive early, assertive, multi-modal nutritional care that is tailored to their needs and goals.

## 6. Research Gaps and Solutions 

COVID-19 is a disease with distinctive and wide-ranging features. Many patients severely affected by the disease had underlying poor metabolic health. Good quality trials are required that investigate the impact of individualised nutrition, including medical nutrition and physical therapy, on recovery and the disease trajectory. These should evaluate the clinical parameters and what matters to patients (patient-related outcome measures). The effectiveness of the available nutritional treatment strategies requires further evaluations through high-quality trials, the results of which need to be systematically reviewed over time. OD and malnutrition are of importance in COVID-19 patients, and the simultaneous management of the two conditions must be proactive, aggressive, universal and start upon admission to the emergency room. The pathophysiology and natural history of COVID-19 OD is still not known, and research is needed to understand if this condition will be part of the post-COVID-19 syndrome. To help embed the continuity of nutritional care at each stage of the patient journey, research should focus on evaluating the most effective solutions to overcome the breakpoints in nutritional care, facilitate strategies for patient self-management and ensure that nutritional care is monitored and evaluated effectively.

## 7. Conclusions

Following hospitalisation for COVID-19, many patients are malnourished and can become sarcopenic, adversely affecting their recovery, physical function and quality of life. Many of them also present with OD. Nutritional and physical therapy as part of a comprehensive multi-disciplinary rehabilitation model of care has the potential to improve their clinical outcome. To achieve this, nutritional support must be assertive, multi-modal, individualised to patients’ needs and maintained across the continuum of care, from hospital admission to discharge at home, until malnutrition and sarcopenia have been resolved and physical function restored. Learning from managing patients with COVID-19 across the continuum of care should be applied to other patient groups who experience acute severe illness.

## Figures and Tables

**Table 2:** Multi-modal nutritional therapy.

Multi-Modal Nutritional Therapy
Multi-modal nutritional therapy means the use of multiple methods employed by the multi-disciplinary team and based on the individual needs and goals identified during nutritional assessment. A combination of nutritional interventions, e.g., dietary counselling, food fortification, food texture modification, thickened fluids, oral nutritional supplements, enteral or parenteral nutrition, should be used, depending on patient needs. Multi-modal can also include the use of specific target nutrients, e.g., vitamin D, protein and omega-3 fatty acids, where indicated, for example, in muscle-targeted nutritional therapy. Nutritional interventions can be combined with other treatment modalities, e.g., physical activity, exercise and/or psychological support, to help patients achieve the outcomes that matter to them.

## Data Availability

Not applicable for review articles.
